# Biodiesel Production by *Aspergillus niger* Lipase Immobilized on Barium Ferrite Magnetic Nanoparticles

**DOI:** 10.3390/bioengineering3020014

**Published:** 2016-05-12

**Authors:** Ahmed I. El-Batal, Ayman A. Farrag, Mohamed A. Elsayed, Ahmed M. El-Khawaga

**Affiliations:** 1 Drug Radiation Research Department, National Center for Radiation Research and Technology (NCRRT), Atomic Energy Authority, Cairo 11371, Egypt; 2 Botany and Microbiology Department, Faculty of Science, Al-Azhar University, Cairo 11371, Egypt; dardear2002@yahoo.com; 3 Chemical Engineering Department, Military Technical College, Cairo 11371, Egypt; aboelfotoh@gmail.com (M.A.E.); ahmedelkhwaga15@gmail.com (A.M.E.-K.)

**Keywords:** biodiesel, *Aspergillus niger*, lipase immobilization, barium ferrite magnetic nanoparticles, fatty acid methyl ester

## Abstract

In this study, *Aspergillus niger* ADM110 fungi was gamma irradiated to produce lipase enzyme and then immobilized onto magnetic barium ferrite nanoparticles (BFN) for biodiesel production. BFN were prepared by the citrate sol-gel auto-combustion method and characterized by transmission electron microscopy (TEM), X-ray diffraction (XRD), Fourier transform infrared (FTIR) and scanning electron microscopy with energy dispersive analysis of X-ray (SEM/EDAX) analysis. The activities of free and immobilized lipase were measured at various pH and temperature values. The results indicate that BFN–Lipase (5%) can be reused in biodiesel production without any treatment with 17% loss of activity after five cycles and 66% loss in activity in the sixth cycle. The optimum reaction conditions for biodiesel production from waste cooking oil (WCO) using lipase immobilized onto BFN as a catalyst were 45 °C, 4 h and 400 rpm. Acid values of WCO and fatty acid methyl esters (FAMEs) were 1.90 and 0.182 (mg KOH/g oil), respectively. The measured flash point, calorific value and cetane number were 188 °C, 43.1 MJ/Kg and 59.5, respectively. The cloud point (−3 °C), pour point (−9 °C), water content (0.091%) and sulfur content (0.050%), were estimated as well.

## 1. Introduction

Biodiesel is defined as the fatty acid alkyl monoesters derived from renewable feed stocks such as vegetable oils and animal fats [1]. Biodiesel is an efficient, clean and 100% natural energy alternative to petroleum-based fuels. Biodiesel fuel has many advantages; it is safe for use in all conventional diesel engines, offers the same performance and engine durability as petroleum diesel fuel and is non-flammable and non-toxic. In addition. It reduces tailpipe emissions, visible smoke, noxious fumes, and odors [2]. Biodiesel is better than diesel fuel in terms of sulfur content, flash point, aromatic content and biodegradability [3]. The simple reaction to convert vegetable oil to biodiesel is called transesterification, in which an alcohol and a catalyst are mixed with oil in order to “crack” the oil into esters and glycerol. During this process, the catalyst allows the alcohol to react successively with triglyceride to produce esters and glycerol, as shown in Equation (1). Where, the heavier portion of the reaction medium, glycerol, falls out of the mixture, leaving methyl esters. On the other hand, the methyl esters are purified by distillation. The distilled product, methyl esters of fatty acids, is known as the biodiesel [4]. There are three steps involved in the transesterification of triglyceride (TG) into methyl esters (ME), with the formation of intermediates diglyceride (DG) and monoglyceride (MG), resulting in the production of three moles of ester and one mole of glycerol, as shown in stepwise reactions Equations (2)–(4) [5].


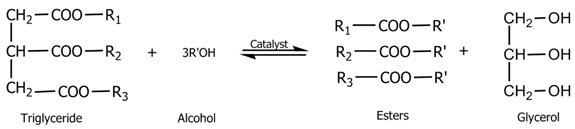
(1)


Triglyceride + ROH ⇌ Diglyceride + RCOOR
(2)

Diglyceride + ROH ⇌ Monoglyceride + RCOOR(3)

Monoglyceride + ROH ⇌ Glycerol + RCOOR
(4)

A catalyst is usually used to improve the reaction rate and yield. Because the reaction is reversible, excess alcohol is used to shift the equilibrium to the products side. Lipases can catalyze this reaction [6].

Lipases (triacylglycerol acyl hydrolases, EC 3.1.1.3) are largely used as biocatalysts in biotechnology and modern chemistry [7]. These enzymes are also catalyzing several reactions like esterification [8], transesterification, acidolysis, interesterification, alcoholysis, aminolysis, oximolysis, thiotransesterification and ammoniolysis in anhydrous organic solvents [9]. Lipase is widely found in animals, plants and microorganisms [10]. Nowadays, most lipases produced commercially are currently obtained from fungi and yeasts. Fungal lipases have received attention because of their potential use in food processing, pharmaceuticals, cosmetics, oils/fats degradation, detergents and the leather industry [11]. Industrial applications of lipase are limited because it has poor stability, is easily inactivated, and is difficult to separate from the reaction system for reuse. In order to use lipase more economically and efficiently in reaction systems, its activity and operational stability need to be improved by an appropriate choice of immobilization process [12]. Immobilization of enzymes is a well reported technology that allows application of enzymes in many biocatalyzed processes, like in lipase-mediated biodiesel production. This process provides significant advantages, such as reuse of the biocatalyst, easier product recovery, and it frequently enhances the enzyme resistance against inactivation by different denaturants, providing more stable catalysts [13]. Besides, the enzyme immobilization onto magnetic supports such as nanosized magnetite particles allows an additional characteristic compared to other conventional support materials, such as the selective and secure enzyme recovery from the medium under the magnetic force. Hence, there is no need for expensive liquid chromatography systems, centrifuges, filters or other equipment [14]. In this study, the lipase from *Aspergillus nigerADM110* was immobilized onto the magnetic Barium Ferrite Nanoparticles (BFN) under different experimental conditions and characterized. The biodiesel production was carried out and analyzed by GC-MS.

## 2. Materials and Methods

### 2.1. Chemicals

All the media components were purchased from Oxoid and Difco. Chemicals and reagents, including olive oil used, were purchased from Sigma-Aldrich (St Louis, MO, USA), were of analytical and HPLC grade, and were used without further purification. *Aspergillus niger ADM110* strain was from Drug Radiation Research Department (NCRRT) [15].

### 2.2. Lipase Production and Purification

Extracellular lipase was obtained from irradiated *Aspergillus niger ADM110* by submerged production using the optimized medium containing olive oil 5% as the most efficient carbon source and yeast extract 5.0% as the most suitable nitrogen source for the lipase biosynthesis. The fermentation was carried out in 100 mL of sterile production broth seeded with 1.0 mL (4.60 × 10^7^) of spore inoculum and incubated at 25 °C, pH 7, 200 rpm agitation speed and for 72 h. *Aspergillus nigerADM110* was subjected to 0.14 kGy of gamma radiation to improve its lipolytic potential [15]. Crude lipase was obtained by centrifuging the culture broth at 10,000 rpm for 10 min at 4 °C. The lipase purification was carried out by ammonium sulfate precipitation followed by dialysis against 0.05 M sodium phosphate buffer solution (pH 7.2). Extracellular lipase activity was estimated by photometric assay [16].

### 2.3. Synthesis of Hexagonal Magnetic Barium Ferrite Nanoparticles (BNF)

Barium ferrite nanoparticles were prepared by the citrate sol-gel auto-combustion method [17,18]. Barium nitrate Ba (NO_3_)_2_, ferric nitrate Fe (NO_3_)_3_·9H_2_O and citric acid were taken as starting materials. The nitrates were dissolved in the minimum amount of de-ionized water to get a clear solution in the molar ratio 1:10. An aqueous solution containing 12.5 moles of citric acid was mixed with both nitrate solutions (metals: citric acid, 1:3). After complete dissolving of all starting materials, an appropriate amount of ammonia solution was added dropwise with agitation until the pH of the solution reaches 7.0 giving a clear dark-green solution. The resulting solution was then heated in a drying oven at 85 °C for 3 h followed by heating at 110 °C until the transparent solution turned to viscous gel. The viscous gel was then heated at 150 °C until foaming occurred. The foamy gel was maintained at 150 °C until spontaneous ignition occurred. The combustion reaction was completed within a few minutes, and brown ash was formed. The fine powder was calcined (pre-sintered) in the calcination oven at 450 °C for 3 h to remove carbonic materials and then the temperature was increased to 850 °C for 4 h for the final formation of barium ferrite nanoparticles.

### 2.4. Immobilization of Lipase on BFN Particles

For the immobilization of lipase, 200 mg of BFN particles were added to 0.85% (*w*/*v*) sodium chloride solution containing lipase. The mixture was incubated for 4 h using an overhead stirrer. After washing twice with 0.85% (*w*/*v*) sodium chloride solution, the immobilized lipase was separated by magnetic decantation of the supernatants and stored at 4 °C. The amount of protein binding to the BFN particles was determined by measuring the protein concentration of the lipase solution and the supernatant by the Lowry method [19].

### 2.5. Assay of Enzyme Activity

The assay was modified from that described by Pencreac’h *et al.* [20]. The assay mixture contained 90 μL of 8.25 mM *p*-nitrophenyl palmitate in isopropanol and 810 μL of 50 mM Tris-HCl, pH 8.0, with 0.5% (*w*/*v*) Triton X-100 and 0.12% (*w*/*v*) Arabic gum. The mixture was then preheated to 40 °C. To initiate the reaction, 100 μL of lipase solution or suspension of immobilized lipase diluted to the appropriate concentration with 1% (*w*/*v*) bovine serum albumin was added. The change in absorbance at 410 nm was monitored for 5 min at 40 °C using a thermostated spectrophotometer (UV-1800, Shimadzu, Kyoto, Japan). The activity was calculated from the difference in absorbance between 2 and 5 min with a standard curve for the hydrolysis product, *p*-nitrophenol. One activity unit was defined as the production of 1 μmol of *p*-nitrophenol per min at 40 °C. Measurements were performed in triplicate.

After the reaction, BFN particles were separated magnetically and recycled. The specific activity of the lipase was defined in this study as the enzyme activity (unit) divided by the protein content and expressed as U/mg protein. Relative specific activity was calculated by dividing the specific activity of the immobilized lipase by that of the free lipase [21].

### 2.6. Biodiesel Production Using the Transestrification Process

Transesterification is the reaction of vegetable oil or animal fat with an alcohol, in most cases methanol, to form esters and glycerol. However, before entering the transesterification reactor, the FFA (Free Fatty Acids) content and humidity must be removed, to avoid the production of soap. The transesterification reaction is affected by (molar ratio of glycerides to alcohol, the amount of catalyst, reaction temperature, reaction time and shaking speed) [22].

The yield of the transesterification processes was calculated as a sum of the weight of FAME (fatty acid methyl ester) produced to the weight of cooking oil used, multiplied by 100. The formula is given as:

$$\text{Yield of FAME}=\frac{Weight\;of\;fatty\;acid\;methylester}{Weight\;of\;fat\;used}\;\times \;100\%$$

#### 2.6.1. Gas Chromatography Analysis of WCO (Waste Cooking Oil)

GC analysis of waste cooking oil was carried out on a GC system Agilent Technologies 7890A (Santa Clara, USA) equipped with FID, split/splitless injector and Agilent 7693 A automated liquid sampler. Column: HP INNOWAX, 30 m × 0.32 mm ID, 0.25 μm film thickness. Temperature program of the oven: initial temperature 210 °C for 9 min, rate 20 °C/min to 230 °C, 10 min. Detector temperature: 300 °C, injector temperature: 250 °C. Carrier gas: He, column flow 1.5 mL/min, split ratio 1:80. Hydrogen flow 40 mL/min, air flow 400 mL/min, make-up gas (nitrogen) 40 mL/min. Injection volume 1 μL. ChemStation for GC was used for instrument control, data acquisition and data analysis [23].

#### 2.6.2. Fatty Acid Methyl Esters (FAMEs) Analysis (Biodiesel Products)

Five hundred milliliters of the reaction mixture were mixed with 1.0 mL isooctane for two min. Following centrifugal separation, the upper organic layer was collected and washed twice with distilled water and dried over anhydrous Na_2_SO_4_. The solvent was dried under N_2_ steam and dissolved in 0.25 mL of CH_2_Cl_2_. The previous GC condition of WCO analysis was applied. The prepared FAMEs were then analyzed using particular fatty acid methyl ester standards (methyl palmitate, methyl stearate, methyl oleate, methyl linoleate, and methyl linolenate; Sigma-Aldrich).

### 2.7. Analytical Methods

The size and morphology of the magnetic nanoparticles were assessed by transmission electron microscopy (TEM) (JEOL, Peabody, MA, USA). The X-ray diffraction (XRD) (Rigaku Corporation, Austin, TX, USA) was performed to check the crystallinity of the magnetic nanoparticles. SEM/EDAX analysis (JEOL, Peabody, MA, USA), and TEM were performed for characterization of the magnetic nanoparticles.

### 2.8. Statistical Analysis

The analysis of data was carried out according to [24]. Means were compared using the least significant difference (LSD at the 5% level) and Duncan’s multiple range tests at the significance *P* = 0.05.

## 3. Results and Discussion

### 3.1. Characterization of BFN Particles

The result shows the production of the hexagonal phase with a high overall agreement between the entry database and the sample diffraction pattern. The XRD pattern illustrates the formation of 92.7% weight percent hexagonal barium ferrite and 7.3% some other phases, like BaFe_2_O_4_ (cubic phase), BaO and Fe_2_O_3_. TEM analysis of the prepared barium ferrite shows the formation of spherical nanoparticles with average particle size diameter ranging from 8 to 25 nm with some unidentified amorphous phases. According to the structure of hexagonal barium ferrite, it has exact element weight ratios of (Ba: Fe: O) equal to (12.3: 60.4: 27.3). By comparing this value with the prepared one, the result was found to be approximately the same by EDAX analysis using SEM.

The prepared BaFe_12_O_19_ hexaferrite particles were characterized via Fourier transformed Infrared spectra (FTIR) Shimadzu FTIR-8400S. The room temperature infrared spectra of prepared samples was recorded in mid-IR range, 4000 cm^−1^ to 400 cm^−1^. A few milligrams of BaFe_12_O_19_ hexaferrite particles were mixed with anhydrous KBr powder and made in the form of a pellet for the measurements.

**Figure 1:**
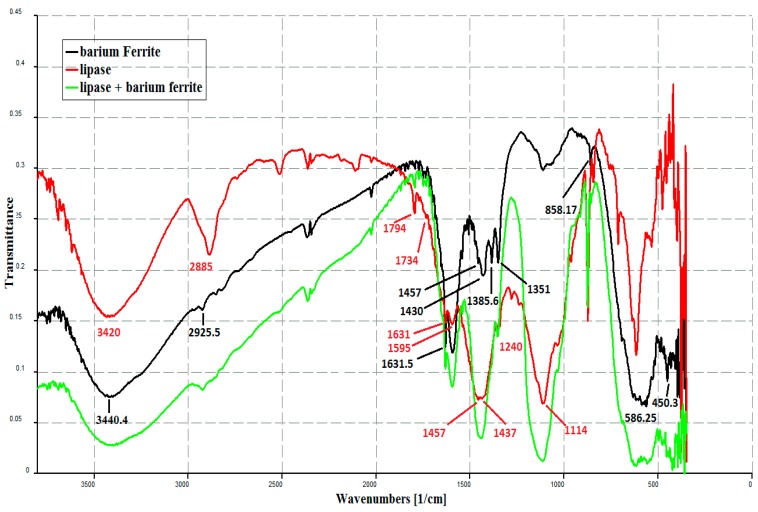
FTIR spectra of: (**a**) free *Aspergillus niger ADM110* lipase enzyme; (**b**) Barium ferrite magnetic nanoparticles; (**c**) Immobilized lipase enzyme on barium ferrite magnetic nanoparticles.

Figure 1a shows FTIR spectra of BaFe_12_O_19_ hexaferrite particles. The main absorption bands at 3440.4 cm^−1^ are attributed to the O–H stretching, while the characteristic band at 1631.5 cm^−1^ results from the anti-symmetrical and symmetrical stretching vibration bands of COO^−^ related to citric acid [25]. The absorption bands at 2925.5 cm^−1^ were due to OH vibrations. The other bands are at 1457, 1430, 1385.6 and 858.17 cm^−1^, corresponding to the stretching and bending vibrations of C=O, H–C–H, C–H and C–C respectively. The characteristic absorption bands between 586.25 and 450.3 cm^−1^ are assigned to the vibration of the bond between the oxygen atom and the metal ions (F–O), confirming the formation of hexaferrite and corresponding to vibrations of the tetrahedral and octahedral sites for BaFe_12_O_19_. The Fe–O stretching vibration band of the bulk magnetite is usually at 570 cm^−1^ and the band shifted to high wave numbers because of the finite size of the nanoparticles. The bands located at 1351 cm^−1^ and 858.17 cm^−1^ are associated with the N–O stretching vibration and bending vibration of NO_3_^−^ [26]. The notable bands at about 1385 and 1430 cm^−1^ are attributed to nitrate ion and barium carbonate, respectively.

Figure 1b shows the FTIR spectrum of lipase from *Aspergillus nigerADM110*. There are many signals characteristic of different functional groups of the enzyme. They include a typical band at 2885 cm^−1^ assigned to the stretching C–H vibrations of –CH2 and –CH3 groups. A broad and intense band at 3420 cm^−1^ was assigned to the stretching vibrations of –OH group, and a band of the –N–H stretching vibrations, masked by the former one [27]. The characteristic signals coming from the stretching vibrations of carbonyl groups around 1734 cm-1 and stretching vibrations of ≡C–O– appear in the spectrum at 1240 cm^−1^.

Figure 1c shows the FTIR spectrum of immobilized lipase on barium ferrite magnetic nanoparticles. It was evident that the characteristic bands of protein (*i.e.*, lipase) at 1794 for Amide I and 1457–1437 cm^−1^ for Amide II with characteristic band 1114 cm^−1^ for coil alpha helix. Furthermore, 1631, 1595 cm^−1^ were present in pure lipase and the lipase-bound (barium ferrite nano) Fe_3_O_4_ nanoparticles, confirming the binding of lipase to BaFe_12_O_19_ nanoparticles by covalent immobilization. The strong characteristic bands of proteins for the lipase-bound BaFe_12_O_19_ should be owing to the high enzyme loading [28]. In addition, it is noted that a characteristic band of 1385.6 cm^−1^ was observed in naked Fe_3_O_4_ nanoparticles. After binding of lipase, this characteristic band disappeared in lipase binding barium ferrite nano. Also, bands at 2885, 2515 and 2111.0 cm^−1^ of lipase disappeared in binding lipase on nanoparticles. Thus, it was suggested that the binding was accomplished via the reaction between the amine group of BaFerrite nanoparticles and the carboxyl group of lipase and confirmed the enzyme immobilization.

### 3.2. The Activity of the Lipase Immobilized on BFN Particles

It is expected that the binding has been taking place between the hydrophobic particle of barium ferrite and the hydrophobic area of the lipase via interfacial activation.

Various initial lipase concentrations (0.2–2 mg/mL) were tested for immobilization experiments for determining the optimum enzyme loading. As shown in Table 1, the amounts of lipase immobilized increased significantly with increasing the initial lipase concentration, and the activities attained a maximum value at an initial lipase concentration of 1.0 mg/mL. The lipase activity is, however, slowly decreased when the initial lipase concentration was above 1.0 mg/mL. The excessive enzyme loading is known to hinder the substrate conversion due to the increased protein–protein interaction [29]. In the rest of the experiments, the initial lipase concentration was maintained at 1.0 mg/mL.

**Figure 2:**
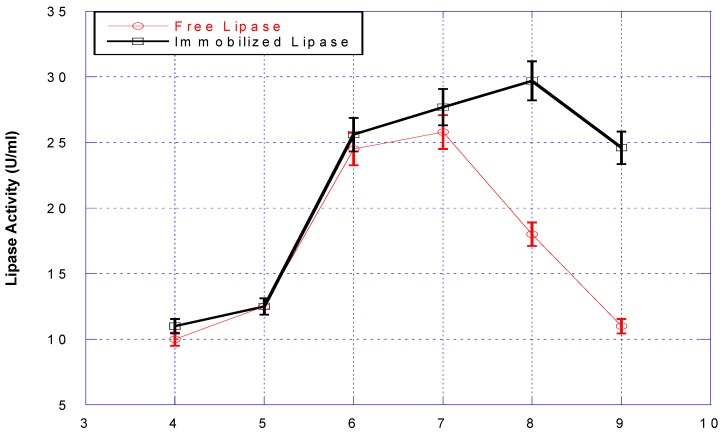
Effect of pH on the activity of free (**red line**) and immobilized (**black line**) lipases.

Activities of free and immobilized lipase were measured at various pH and temperatures values. The pH of the free and the immobilized lipase activities was compared and shown in Figure 2. The optimum pH of the immobilized lipase was 8.0, whereas that of the free one was 7.0. Also, the immobilized lipase showed higher activity than the free one, especially at pHs higher than 7.0.

**Figure 3:**
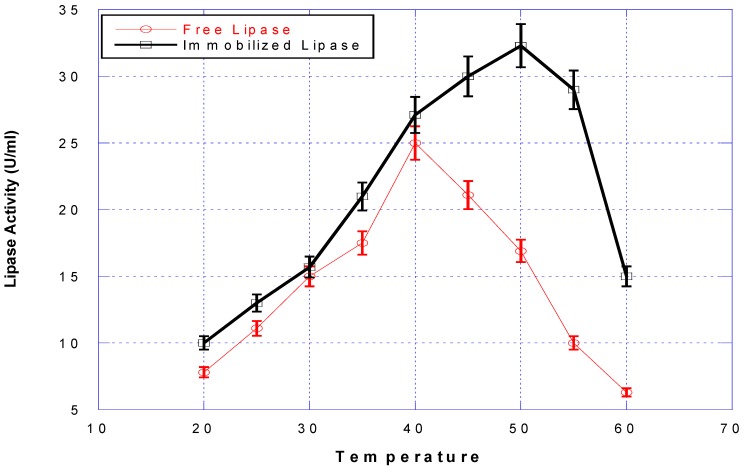
Effect of temperature (°C) on the activity of free (**red line**) and immobilized (**black line**) lipases at pH 8.0.

The optimum pH value of free lipase shifted one unit to the alkaline region after binding on the support. It would therefore appear that lipase active sites may well be occluded in some of the composites. Also, the BFN to which the lipase is immobilized retards the denaturing effect of increased pH and/or temperature, which is exhibited by the shift in the optimal pH/temperature for activity. Previous studies [30] have suggested that upon immobilization, the active site becomes more exposed to solvent than that in the folded-dissolved lipase form. Activities of free and immobilized lipase were measured at various temperatures and the results are shown in Figure 3.

The optimum temperature of the immobilized lipase was 50 °C, whereas that of the free one was 40 °C. It can be concluded that the immobilization using BFN particles enhanced the thermal stability of lipase, which might be caused by multipoint attachment of the lipase to the support and/or by the hydrophobic interaction [31].

### 3.3. Recyclability of BFN-Lipase

After the reaction was complete, the immobilized lipase can be easily separated by a magnet. The results indicate that BFN-Lipase (5%) can be reused in biodiesel production without any treatment with 17% loss of activity after 5 cycles and a 66% loss in activity in the sixth cycle, with the activity of 29, 26, 25, 25 and 24 U/mL around the five cycles, respectively. Then the activity was decreased to 10 U/mL after the 6th cycle.

Therefore, no appreciable inhibition due to oil, glycerol or product was detected. Importantly, no aggregation of the particle was observed. The behavior of the prepared sample indicates that BFN particles prevent the undesirable hydrophilic interactions (glycerol and methanol adsorption, protein aggregation, particle aggregation) which are likely to occur in the hydrophobic oils. The results presented in Table 2 show the fatty acid profile of WCO and its biodiesel fatty acid methyl ester.

### 3.4. Production of Biodiesel Using the Transesterification Process

One liter sample size of waste cooking oil was heated to 60 °C to remove any free water and allowed to settle for 24 h before reacting with methanol.

#### 3.4.1. Effect of Methanol/Waste Cooking Oil Molar Ratio on Biodiesel Production

Stoichiometrically, three moles of methanol are required for each mole of triglyceride, but in practice, a higher molar ratio is needed to drive the reaction towards completion and produce more fatty acid methyl esters (FAMEs) as products.

In this study, biodiesel was obtained under transesterification conditions as follows: reaction temperature, 45 °C; catalyst amount, 5.0 wt %; reaction time, 4 h. The effect of methanol: oil molar ratio increases from 1:1 to 4:1, as listed in Table 3, in which the biodiesel yield increases as the molar ratio increases from 1:1 to 4:1. The maximum biodiesel yield of 90% is obtained when the molar ratio is very close to 4:1. However, beyond the molar ratio of 5:1, the excessively added methanol has no significant effect on the production yield. In contrast, when the amount of methanol is over 4:1, glycerol separation becomes more difficult, thus decreasing the biodiesel yield. Based on this, the optimum molar ratio of methanol to oil is 4:1.

#### 3.4.2. Effect of Enzyme Concentration Percentage on Biodiesel Production

The effect of catalyst dosage was investigated with the mass ratio of immobilized lipase catalyst to waste cooking oil varying within the range of 3.0%–20.0%, under otherwise identical conditions as reaction temperature, 45 °C; methanol/oil, 3:1; reaction time, 4 h. The biodiesel yield was found to increase with increasing catalyst dosage, and the maximum was obtained at the dosage of 20% with the value being 93% at 4 h Table 3. It should be noted that we could not continue increasing catalyst concentration over 20% due to the high cost of immobilized lipase. So, we can deduce that 89% of biodiesel obtained at 5% enzyme concentration is the superior result.

#### 3.4.3. Effect of Reaction Temperature on Biodiesel Production

In this part of the experiments, the reaction temperature was varied within a range of 15–55 °C. Transesterification conditions: methanol/oil, 3:1; catalyst amount, 5.0 wt % (of the feed mass of waste cooking oil, similarly from now on); reaction time (4 h). The results listed in Table 3 indicated that the reaction rate was slow at low temperatures and the biodiesel yield was only 55% at 15 °C after 4 h. The biodiesel yield increased with the increase of the reaction temperature to nearly 91% at 45 °C, but at higher temperatures (*T* > 55 °C), the methanol was vaporized and formed a large number of bubbles in the interface, which inhibited the increase of biodiesel yield, then the yield of biodiesel decreased significantly. Thus, the optimum reaction temperature was 45 °C.

#### 3.4.4. Effect of the Reaction Time on Biodiesel Production

The reaction time tells the fastness of the transesterification process of a particular feedstock. The results in Table 3 indicated that the ester content increased with reaction time from 2 h onward and reached a maximum at a reaction time of 4 h at reaction temperature, 45 °C; methanol/oil, 3:1; reaction time, 4 h; catalyst amount, 5.0 wt % of the feed mass of waste cooking oil, and then remained relatively constant with increasing further the reaction time. An extension of the reaction time from 4 to 6 h had no significant effect on the conversion of triglycerides but led to a reduction in the product yield. Because longer reaction enhanced the hydrolysis of esters (reverse reaction of transesterification), this resulted in the loss of esters.

#### 3.4.5. Effect of Shaking Speed on Biodiesel Production

The agitation rate improves the mixing during the transesterification process. It increases the intact area between oils and immobilized lipase enzyme methanol solution and facilitates the initiation of the reaction.

Transesterification conditions: methanol/oil, 3:1; catalyst amount, 5.0 wt % (of the feed mass of waste cooking oil, similarly hereinafter); reaction time, 4 h and at 45 °C. In methanolysis conducted with different rates of stirring, such as 100, 200, 300, 400 and 500 revolutions per minute (rpm), it was observed that the reaction of methanolysis was practically incomplete at 100 rpm and only exhibited a yield of 43%, as in Table 3. The yield was increased when the mixing intensity was accelerated up to 400 rpm.

### 3.5. Physicochemical Properties of the Used Cooking Oil and Its Corresponding Production of Biodiesel

#### 3.5.1. Kinematic Viscosity

Viscosity refers to a fluid’s resistance to flow at a given temperature. Fuel that is too viscous can hinder the operation of an engine. Kinematic viscosity measures the ease with which a fluid will flow under force. It is different from absolute viscosity, also called dynamic viscosity. Kinematic viscosity is obtained by dividing the dynamic viscosity by the density of the fluid [32]. Kinematic viscosity allows comparison between the engine performances of different fuels, independent of the density of the fuels. Two fuels with the same kinematic viscosity should have the same hydraulic fuel properties, even though one fuel may be denser than the other.

The kinematic viscosity of the used waste cooking oil was 60.0 cSt and its corresponding biodiesel was 5.83 cSt (Table 4).

#### 3.5.2. Density at 15 °C

The density of biodiesel is used to judge the homogeneity of biodiesel. This property is important, mainly in airless combustion systems because it influences the efficiency of atomization of the fuel [33]. The result obtained (Table 4) showed that the methyl ester produced in this study had a density of 0.850 g/mL, which falls in the range 0.82–0.87 g/mL, specified according to Egyptian diesel oil.

#### 3.5.3. Distillation at Atmospheric Pressure

**Figure 4:**
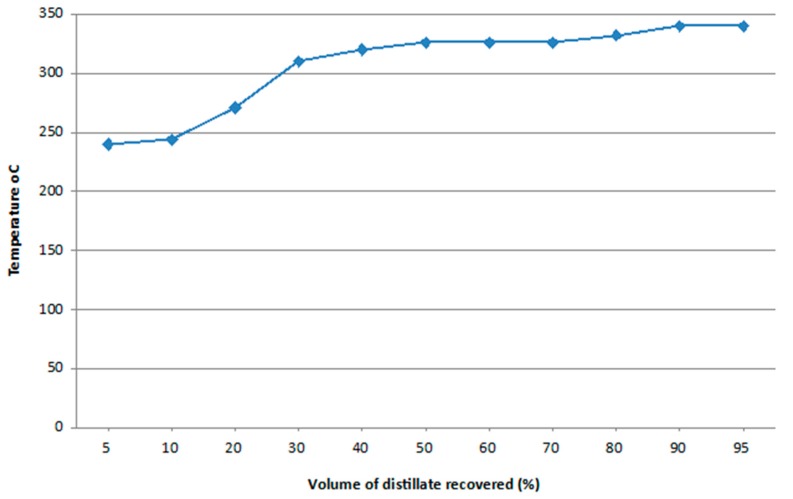
Relationship between volume of distillate recovered and temperature °C.

Distillation is used to determine the boiling range of the biodiesel product quantitatively. Even though distillation at the atmospheric pressure provides some clues or information about the properties of methyl esters such as the boiling range of the biodiesel product, distillation at the reduced pressure (vacuum distillation) is required because the biodiesel will thermally decompose using atmospheric distillation. The plot pattern of the volume of distillate recovered *versus* temperature in Figure 4 indicates this fact. The decrease in temperature during the distillation process shows the decomposition of methyl ester which results in the formation of low boiling molecular substances.

#### 3.5.4. Flash Point (FP)

The flash point temperature is the measure of the fuel to form a flammable mixture with air. It is only one of many properties which must be considered in assessing flammability hazard of material. For biodiesel, a flash point of below 130 °C is found to be out of specification (ASTM D 6751, 2009). The measured flash point of this biodiesel was 188 °C (Table 4), indicating very small or negligible methanol levels in the biodiesel. This high flash point can prevent auto ignition and fire hazard at a high temperatures during transportation and storage periods.

#### 3.5.5. Heat of Combustion/Calorific Value

Gross heat of combustion (HG) is a property proving the suitability of using fatty compounds as diesel fuel. The calorific value of the obtained biodiesel; 43.1 MJ/Kg is an acceptable value (Table 4). Although it is lower than the Egyptian standards for petrodiesel (44.3 MJ/Kg), it is higher than that of the European standard for biodiesel (32.9 MJ/Kg).

#### 3.5.6. The Cetane Number

The cetane number (CN) is a dimensionless descriptor of the ignition quality of a diesel fuel (DF) [34]. It is a prime indicator of DF quality. It is a measure of how easily the fuel will ignite in the engine. The CN is related to the time that passes between injection of the fuel into the cylinder and the start of ignition. Because of the higher oxygen content, biodiesel has a higher cetane number compared to petroleum diesel. The cetane index of waste cooking oil from the experiment was found to be 59.5 (Table 4). The cetane number of methyl esters of rapeseed oil, soybean oil, palm oil, lard and beef tallow were found to be 58, 53, 65, 65 and 75, respectively [35]. Among these biodiesel feed stocks, beef tallow has the highest cetane number. The higher cetane number indicates, the higher engine performance of beef tallow compared to other fuels, resulting in the lower emission of all pollutants other than oxides of nitrogen (NO*x*). As beef tallow has the higher amount of saturated fatty acids, the increase in the saturated fatty acids content positively enhanced the cetane number of biodiesel. The obtained data revealed the higher cetane number, 59.5 compared to Egyptian petrodiesel standards of 55, but within the recommended universal biodiesel standards (min 47).

#### 3.5.7. Cloud Point (CP) and Pour Point

The cloud and pour points are also important properties of biodiesel fuel. The cloud point is the temperature at which crystals first appear in the fuel when cooled [36]. At temperatures below CP, larger crystals fuse together and form large agglomerates that can restrict or cut off flow through fuel lines and filters and cause start-up and performance problems the next morning [37]. The temperature at which crystal agglomeration is extensive enough to prevent free pouring of fluid is determined by measurement of its pour point [38]. These properties are related to the use of biodiesel in cold temperatures. The cloud and pour points are (−3 and −9 °C), respectively (Table 4). So the produced biodiesel would be more suitable to cold conditions as compared to petrodiesel.

#### 3.5.8. Water and Sulfur Content

Biodiesel is sulfur free, but this test is an indicator of contamination of protein material and/or carryover catalyst material or neutralization material from the production process (ASTM D4294). Sulfur reduces the function of catalysts and causes SO*x* emissions as in diesel engines and has an adverse effect with the formation of black smoke. Hence, a low level of sulfur, or no sulfur, is beneficial to the performance of the diesel engine due to the lower emissions and the decrease in the levels of corrosive sulfuric acid accumulating in the engine crankcase oil. Sulfur content of biodiesel is from 0.031 to 0.05. The synthesized kind is found to be in this range.

Water can cause corrosion of tanks and equipment, and if detergent is present, the water can cause emulsions or a hazy appearance. Water supports microbiological growth at the fuel/water interfaces in fuel systems. Biodiesel can contain as much as 1500 parts per million of dissolved water [39]. The produced biodiesel is characterized by low water and sulfur content, 0.091% and 0.050% respectively (Table 4), compared with the Egyptian petrodiesel standards, <0.15% and 1.2%, respectively.

#### 3.5.9. Iodine Number

The iodine value is an important measure for the determination of the unsaturation in fatty acids, which is only dependent on the vegetable oil. This property greatly influences fuel oxidation and the deposits formed in the injector of diesel engines [40]. With the increasing iodine value of unsaturated fatty acids, the effect of polymerization is stronger. In this study, the iodine value of the WCO used was 125 mg I_2_/100 g oil, and 102 mg I_2_/100 g oil for the corresponding biodiesel (Table 4). According to EN 14214, the biodiesel must have an iodine value <120 mg I_2_/100 g oil in the sample.

#### 3.5.10. Acid Number

The acid number is the quantity of base, expressed as milligrams of KOH per gram of sample, required to titrate a sample to a specified end point. Acid number determination is an important test to assess the quality of a particular biodiesel. It can indicate the degree of hydrolysis of the methyl ester, a particularly important aspect when considering storage and transportation, as large quantities of free fatty acids can cause corrosion in tanks [41]. The acid value of feedstock and produced methyl esters was 1.90 and 0.182 (mg KOH/g oil), respectively (Table 4). The average percentage decrease of acid value from WCO to the produced biodiesel was about 90.4%, indicating a good transesterification process. This result meets the EN 14214 and D-6751 standards, but it was higher than that of Egyptian petrodiesel standards.

#### 3.5.11. Saponification Value (mg KOH/g Oil)

Saponification value is the quantity of base, expressed as milligrams of KOH per gram of sample, required to saponify one gram of fat or oil. The obtained result (Table 4) indicated that the methyl esters produced had higher saponification value 206 (mg KOH/g oil) than the corresponding oils 201 (mg KOH/g oil).

## 4. Conclusions

Biodiesel production technology is competitive in terms of it being a low-cost and alternative source of energy, which should be not only sustainable but also environmentally friendly. Catalyst immobilization addresses several issues: repetitive and continuous use, localization of the interaction, prevention of product contamination, reduction of effluent problems and material handling, and effective control of the reaction parameters. In this study, we developed novel hydrophobic magnetic nanoparticles (barium ferrite nanoparticles) for lipase immobilization. This allows additional characteristics compared to other conventional support materials, such as selective and easy enzyme recovery from the medium under the magnetic force. The second advantage is that we can reuse the BFN-Lipase without any rinsing treatment for five cycles, with an activity of 29, 26, 25, 25 and 24 U/mL during the five cycles. The activities of free and immobilized lipase were measured at various pH and temperatures values. The optimum pH of the immobilized lipase was 8.0, whereas that of the free one was 7.0, and the optimum temperature of the immobilized lipase was 50 °C, whereas that of the free one was 40 °C. The fatty acid profile of collected waste cooking oil as feedstock for lipase production was determined using GC/MS and showed the presence of oleic, palmitic, stearic, linoleic and linolenic acids. The enzymatic conversion of waste cooking oil to biodiesel (transesterification) was carried out using methyl alcohol. Different factors affecting transesterification reaction, such as methanol/oil molar ratio, reaction temperature, shaking, reaction time and enzyme concentration, were studied. The previous results showed that the use of immobilized lipase enzyme on barium ferrite nanoparticles had high efficiency as a catalyst in the process of biodiesel production from waste cooking oil. Tests also demonstrated that the properties of biodiesel attained were fit as a substitute for diesel in addition to being a friend of the environment.

## Figures and Tables

**Table 1:** Activity of the lipase immobilized on BFN (barium ferrite nanoparticles) particles.

Initial Lipase Concentration (mg/mL)	Activity of Immobilized Lipase (U/mL)	Protein Immobilized (mg/g Support)	Specific Activity (U/mg Protein)
00.2	4.8a ± 0.56	9.0a ± 0.70	6.15a ± 0.10
00.4	12.5b ± 0.35	15.7b ± 0.49	7.30b ± 0.21
00.6	23.9c ± 0.63	24.8c ± 0.42	8.82c ± 0.57
00.8	27.0d ± 0.56	36.5d ± 1.06	9.44de ± 0.39
01.0	30.5f ± 0.77	49.0e ± 0.91	10.10e ± 0.61
01.4	28.6e ± 0.77	57.8g ± 0.56	8.92cd ± 0.26
01.8	28.4e ± 0.98	52.6f ± 0.91	9.75de ± 0.53
02.0	28.5e ± 0.35	52.5f ± 0.56	9.60de ± 0.35
LSD	01.70	03.25	1.01

Note: Different letters indicate significant differences between treatments (Duncan test, *P* ≤ 0.05). Means in each column followed by the same letter are not significantly different.

**Table 2:** Fatty acid profile of WCO and its biodiesel fatty acid methyl ester.

Fatty Acids	Formula	Common Acronym	Methyl Esters	% Composition by Mass
Oleic acid	C_17_H_33_COOH	C18: 0	Methyl oleate	46.5d ± 0.91
Palmitic acid	C_15_H_31_COOH	C16: 0	Methyl palmitate	30.9c ± 0.77
Stearic acid	C_17_H_35_COOH	C18: 0	Methyl strearate	09.0b ± 0.56
Linoleic acid	C_17_H_31_COOH	C18: 2	Methyl linoleate	08.5b ± 0.35
Linolenic acid	C_17_H_29_COOH	C18: 3	Methyl linolenate	05.1a ± 0.28
LSD				02.95

Note: Different letters indicate significant differences between treatments (Duncan test, *P* ≤ 0.05). Means in each column followed by the same letter are not significantly different.

**Table 3:** Factors affecting biodiesel production using transesterification process.

Factors Affecting Biodiesel Production		Biodiesel Yield (%)
Methanol/oil ratio (mole/mole)	1:1	42a ± 1.41
	2:1	65b ± 2.12
	3:1	87d ± 0.707
	**4:1**	**90e ± 1.76**
	5:1	76c ± 0.707
LSD		03.75
Enzyme concentration (%)	3	69a ± 0.707
	**5**	**89b ± 1.06**
	10	91bc ± 1.41
	15	94c ± 0.707
	20	97d ± 1.909
LSD		02.25
Reaction temperature (°C)	15	55a ± 0.494
	25	73b ± 1.41
	35	81c ± 0.636
	**45**	**91d ± 1.41**
	55	75b ± 0.707
LSD		05.95
Reaction time (h)	2	50a ± 0.707
	**4**	**88d ± 1.06**
	6	88d ± 1.41
	8	61c ± 0.848
	10	58b ± 0.707
LSD		03.10
Shaking speed (rpm)	100	43a ± 1.41
	200	52b ± 1.48
	300	71c ± 1.41
	**400**	**88d ± 0.636**
	500	69c ± 1.13
LSD		09.05

Note: Different letters indicate significant differences between treatments (Duncan test, *P* ≤ 0.05). Means in each column followed by the same letter are not significantly different.

**Table 4:** Physicochemical properties of the produced biodiesel.

No.	Characteristics	Result	Unit	Test Method
1	Kinematic viscosity at 40 °C	5.83	mm^2^·s^−1^	ASTM D445
2	Density at 15.5 °C	0.850	g·cm^−3^	ASTM D1298
3	Calorific value	43.1	MJ/Kg	ASTM D-224
4	Total sulfur content	0.050	mass%	ASTM D4294
5	Flash point	188	°C	ASTM D92
6	Pour point	−9	°C	ASTM D97
7	Cloud point	−3	°C	ASTM D2500
8	Cetane number	59.5	—	ASTM D613
9	Water content	0.091	vol%	ASTM D6304
10	Acid number	0.182	mg KOH g^−1^	ASTM D664
11	Distillation temperature (DT)	95% Recovery at 340	°C	ASTM D86
12	Iodine number	102	mg I_2_/100 g oil	ASTM D4737
13	Saponification value	206	mg KOH/g oil	ASTM D 5558
